# Editorial: Longitudinal development of aerobic fitness in children

**DOI:** 10.3389/fpubh.2023.1250256

**Published:** 2023-08-08

**Authors:** Craig Anthony Williams, Amund Riiser, Asgeir Mamen

**Affiliations:** ^1^Children's Health and Exercise Research Centre (CHERC), Faculty of Life and Health Sciences, University of Exeter, Exeter, United Kingdom; ^2^Faculty of Education, Arts and Sports, Western Norway University of Applied Sciences, Sogndal, Norway; ^3^School of Health Sciences, Kristiania University College, Oslo, Norway

**Keywords:** children, aerobic fitness, health, longitudinal development, physical activity

If the only thing constant in children's life is change, then closely following the development of children is of interest. The aerobic fitness and health of children have been investigated in detail for nearly a century now. Aerobic fitness was probably first addressed by Sid Robinson in 1938, and the knowledge from this and other studies has been valuable for making guidelines about the level of activity among children. The advent of simple ECG-like heart rate monitors in the 1980's made it possible to monitor physical activity more objectively. Furthermore, when accelerometers became available, the recommendations for physical activity could now have a more robust scientific base due to better validity and reliability of measuring activity. Importantly such studies, incorporating these advancements in how physical activity can be measured can have both a cross-sectional or longitudinal design, and they are essential to gain further knowledge of aerobic fitness for children and young people.

In this special edition, we present six articles that cover a wide range of topics related to the development of children. Three of the included studies assess the longitudinal development of aerobic fitness in populations of children and adolescents with different geographic, athletic and health backgrounds. The studies demonstrated associations between development of aerobic fitness and biological maturation, participation in organized sports and the COVID-19 pandemic.

In their study of junior rowers de Almeida-Neto et al. from Brazil followed 52 boys and girls with annual performance tests over 3 years. The study demonstrates that advancement in biological maturation, defined from peak height velocity, was positively associated with mean anaerobic power (measured in watts) during indoor rowing (η^2^ *p* > 0.36, *p* < 0.05), and improved performance in rowing competition (η^2^ *p* > 0.35, *p* < 0.05). However, the biological maturation was not associated with maximal oxygen consumption (measured in mL·kg^−1^·min^−1^) (*p* > 0.05). The results were consistent for both sexes.

Järvamägi et al. assessed the development of cardiorespiratory fitness (CRF) in Estonian children during the transition from kindergarten to primary school. They found that children who consistently participated in organized sport performed were better at a 20-m shuttle run test (31.3 ± 13.5 laps,) and had less body fat (21.1 ± 6.3% body fat) at 11.5 years of age compared to children who had never participated in sports clubs (20.7 ± 12.0 laps, 26.1 ± 6.8% body fat).

Raine et al. aimed to assess baseline data from two ongoing clinical trials to determine if cardiorespiratory fitness and fatness were different during COVID-19 relative to before COVID-19. They tested 122 children aged 9.7 years pre COVID-19 and 78 children aged 10.1 years during the COVID-19 pandemic. They reported that pre-COVID-19 children had greater maximal oxygen consumption (43.9 ± 0.7 mL·kg^−1^·min^−1^), compared to children tested during COVID-19 (39.4 ± 0.8 mL·kg^−1^·min^−1^) *p* ≤ 0.001. The results were consistent after adjusting for age and sex. The authors concluded that the results indicate that the COVID-19 pandemic, and behavior changes taken to reduce potential exposure, might have led to lower cardiorespiratory fitness levels in children.

The association between cardiorespiratory fitness and health was also assessed by Souilla et al. from France. As children with congenital long QT syndrome (LQTS) have increased risk of arrhythmic events during exercise it is difficult to balance exercise restrictions vs. promotion of physical activity. However, cardiorespiratory fitness, muscle fitness, and physical activity, have been scarcely explored in children with LQTS. Twenty children with LQTS (12.7 ± 3.7 y) and 20 healthy controls (11.9 ± 2.4 y) performed a maximal cycle ergometer test and used a waist-worn tri-axial accelerometer (Actigraph GT3X). They reported lower maximal oxygen consumption in children with LQTS (33.9 ± 6.2 mL·kg^−1^·min^−1^ compared to healthy controls (40.1 ± 6.6 mL·kg^−1^·min^−1^, *p* = 0.010) and no differences in moderate-to-vigorous physical activity were observed between the groups. The authors concluded that cardiovascular rehabilitation could be of importance for children with LQTS.

Tuan et al. also assessed cardiorespiratory fitness in a specific population of children. They aimed to provide baseline information on cardiopulmonary fitness (CRF) of Taiwanese children and adolescents as most studies evaluating the association between cardiorespiratory fitness, age and sex are performed among western children. The authors recruited 897 children aged 4–18 years into four age categories. They reported that maximal oxygen consumption was higher for boys than girls in all age groups (*p* < 0.05) and that maximal oxygen consumption is strongly and significantly correlated with age in both boys and girls (*r* = 0.9). The authors concluded that the sex-difference in maximal oxygen consumption was observed earlier in children in Taiwan compared to western children.

The last study in the present Research Topic was also performed in the east where Xie et al. explored the relationship between school-age children's fundamental movement skills (FMS), physical fitness levels, and the health-related quality of life (HRQoL) and the mediating role of physical fitness levels between school-age children's FMS and HRQoL. The authors investigated 334 children aged 6–10 years (mean age 8.2 y). Physical fitness was positively associated with HRQoL, physical functioning, social functioning, and school functioning (*r* = 0.244–0.301, *p* < 0.01) and physical fitness mediated FMS, physical functioning [indirect effect = 0.089 (95% CI = 0.01, 0.20)], and school functioning [indirect effect = 0.065 (95% CI = 0.01, 0.15)]. The authors concluded that improved physical fitness may improve the HRQoL of school-age children.

These articles continue to shed light on different and essential aspects of child fitness and hopefully will enhance our understanding of the constantly growing, maturing, and developing human, that of the healthy child and adolescent.

## Author contributions

All authors listed have made a substantial, direct, and intellectual contribution to the work and approved it for publication.

